# NK-cell receptor modulation in viral infections

**DOI:** 10.1093/cei/uxae045

**Published:** 2024-05-20

**Authors:** Marzena Lenart, Magdalena Rutkowska-Zapała, Maciej Siedlar

**Affiliations:** Department of Clinical Immunology, Institute of Pediatrics, Jagiellonian University Medical College, Wielicka, Krakow, Poland; Department of Clinical Immunology, Institute of Pediatrics, Jagiellonian University Medical College, Wielicka, Krakow, Poland; Department of Clinical Immunology, Institute of Pediatrics, Jagiellonian University Medical College, Wielicka, Krakow, Poland

**Keywords:** NK cells, NK-cell receptors, activating receptors, inhibitory receptors, viral infections

## Abstract

Natural killer (NK) cells play a crucial role in controlling viral infections. The ability to kill infected cells without prior immunization, yet being tolerant to self, healthy cells, depends on the balance of germ-line encoded surface receptors. NK-cell receptors are divided into either activating, leading to activation of NK cell and its cytotoxic and pro-inflammatory activity, or inhibitory, providing tolerance for a target cell. The signals from inhibitory receptors dominate and NK-cell activation requires stimulation of activating receptors. In viral infections, NK-cell interaction with infected cells can result in activation, memory-like NK-cell differentiation, or NK-cell exhaustion, which constitutes one of the viral immune evasion mechanisms. All of these states are associated with the modulation of NK-cell receptor expression. In this review, we summarize the current knowledge of NK-cell receptors and their role in viral infection control, as well as the alterations of their expression observed in acute or chronic infections. We present recently discovered SARS-CoV-2-mediated modulation of NK-cell receptor expression and compare them with other human viral infections. Finally, since modulation of NK-cell receptor activation gives a promising addition to currently used antiviral therapies, we briefly discuss the clinical significance and future perspective of the application of agonists or antagonists of activating and inhibitory receptors, respectively. In sum, our review shows that although much is known about NK-cell receptor biology, a deeper understanding of NK-cell receptors role in viral infections is still needed.

## Teaser

This review summarizes recent knowledge on NK cell receptor alterations mediated by viral infections, along with the mechanisms responsible for this modulation. We present recently discovered SARS-CoV-2-mediated modulation of NK cell receptor expression and compare them with other human viral infections.

## Introduction

Natural killer (NK) cells are known for their ability to kill altered, infected, or malignant cells, without prior immunization, yet being tolerant to self, healthy cells. This phenomenon is largely controlled by the balance of germ-line encoded NK-cell receptors. The important role of NK cells and their receptors in antiviral immunity and viral evasion has been known for years, yet the recent SARS-CoV-2 pandemic shed new light on these topics. SARS-CoV-2 was proved to downregulate NK-cell functions by mediating inhibition of activating NK-cell receptors, i.e. NKG2D [1] and DNAM-1 [2], as well as activation of their inhibitory receptors, such as NKG2A [3] or CD161 [4]. The latter observations, particularly, open new therapeutic possibilities similar to checkpoint molecules-based therapies in the diseases associated with T-cell exhaustion. Moreover, the observations regarding less known NK-cell receptors, such as CD161 [4] or Siglec-9 [5], indicate that there is still a need to study NK-cell receptor biology and functions. Thus, in this review, we summarize the current knowledge of the role of NK-cell receptors in viral infections, and their modulation during acute or chronic viral infections, as well as associated mechanisms of immune evasion. What is more, we briefly discuss the clinical significance and future perspective of the application of the receptors’ agonists or antagonists, in the light of the presence of particular receptors on other lymphocyte populations.

## NK cells and their functions

NK cells are large, granular lymphocytes, constituting approx. 5–20% of lymphocytes in human peripheral blood [6]. NK cells’ major role is to kill altered (infected or neoplastic) cells. The target cell is killed by perforin and granzymes, that are secreted from NK-cell lysosomes, or by activation of apoptosis receptors on its surface, mediated by Fas ligand (FasL), tumor necrosis factor (TNF-α) or TNF-related apoptosis-inducing ligand (TRAIL) expressed by NK cells [7, 8]. NK cells secret a number of pro-inflammatory cytokines and chemokines, such as interferon γ (IFN-γ), TNF-α, interleukin 5 (IL-5), IL-13, macrophage inflammatory protein (MIP-1), granulocyte-macrophage colony-stimulating factor (GM-CSF), and RANTES [9]. These cytokines activate Th1 response, stimulate macrophages, T and B lymphocytes, and upregulate the expression of human leukocyte antigen (HLA) class I molecules [10].

In humans, NK cells are usually divided into two main subsets, basing on their surface expression of CD56 and CD16 markers. In peripheral blood, the prevalent CD56^dim^CD16^bright^ subset, consisting 90% of NK cells, possesses significantly higher cytotoxic activity, expressing higher level of perforin, granzymes, and cytolytic granules [11]. The expression of CD16 (Fc gamma receptor III; FcγRIII) facilitates antibody-dependent cellular cytotoxicity (ADCC) [12]. In contrast, their precursors, CD56^bright^CD16^dim^ cells, which consist of up to 10% of circulating NK cells, show low-cytotoxic activity, yet are the main cytokine producers in response to cytokine stimulation (IL-12 and IL-18), but not in response to target cells [13]. In mice, common NK-cell markers are NK1.1 (NKR-P1C) and NCR1 (NKp46/CD335), as well as CD49b (DX5, Integrin VLA-2α) [14], while murine NK-cell subsets are described on the basis of CD27 and CD11b expression. CD11b^+^CD27^+^ are the most potent effector cells, while more matured NK-cells exhibiting CD11b^+^CD27^−^ phenotype, express high levels of inhibitory Ly49 receptor and unique chemoattractant receptors, i.e. CXCR1 [15].

## NK-cell receptors and their role in NK-cell functions

**Figure 1.**
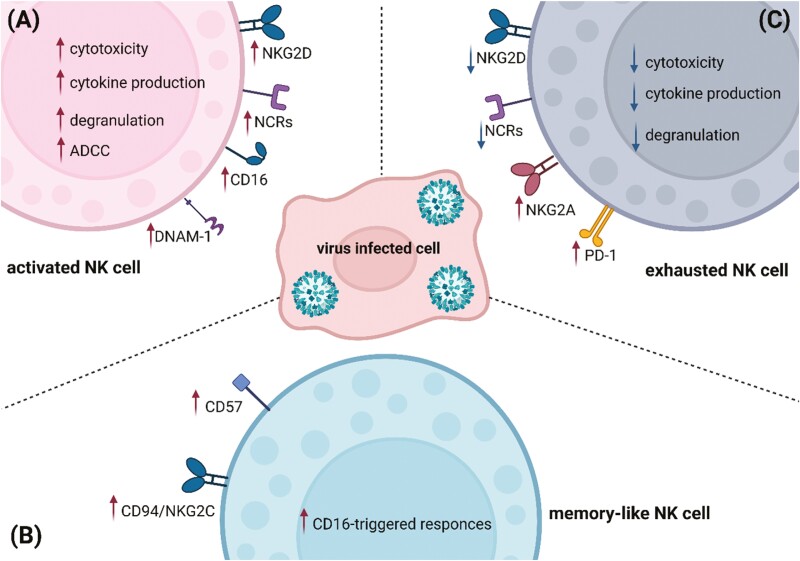
NK-cell interaction with virus infected cell can result in activation, exhaustion, or memory-like NK-cell differentiation, associated with the changes of NK-cell receptor expression. In physiological conditions, NK-cell activates after contact with an infected cell (**A**) Viral infection can result in the development of memory-like NK-cell phenotype (**B**). Viral infection might also mediate NK-cell exhaustion state (C). Created with BioRender.com

NK-cell activation and functions depend on the balance of germ-line encoded surface receptors, which can be categorized as either activating or inhibitory (Fig. 1). Activating receptors recognize their ligands that are expressed on infected or malignant cells. Inhibitory receptors bind self-HLA class I, class I-like molecules, or specific ligands, that are often downregulated on altered cells. It is believed that the balance between activating and inhibitory receptors determines tolerance against healthy cells, yet recognizes altered cells, however, it seems that inhibitory receptors dominate over activating [16]. NK cells’ best-described activating receptors are: activating killer immunoglobulin-like receptors (KIRs), C-type lectin-like receptors (NKG2D, CD94/NKG2C, NKp80), natural cytotoxicity receptors (NCR) (NKp30, NKp44, NKp46), Ig superfamily receptors (DNAM-1), and signaling lymphocytic activation molecule (SLAM) family of receptors (2B4, CD48, NTB-A, and CRACC) [16–18]. NK-cell inhibitory receptors act similarly to T-cell immune checkpoints, regulating antiviral NK-cell functions, by recognition of ligands expressed on infected cells. Best known NK-cell inhibitory receptors are inhibitory KIRs, however, NK cells express a number of non-HLA-specific inhibitory receptors, including CD94/NKG2A, TIGIT, Tim-3, PD-1, and CD161 [19–22]. They can be constitutively expressed on NK cells, such as inhibitory KIRs or CD94/NKG2A, being involved in NK-cell-mediated tolerance of healthy cells, while others, i.e. PD-1, are expressed at a low level on NK cells isolated from healthy donors, yet their expression upregulates in pathological conditions [19, 21].

For proper functioning, NK cells have to integrate signals mediated by both activating and inhibitory receptors. The tolerance of target cells relies on KIRs recognizing normal levels of MHC class I molecules, which phenomena established a concept of “missing-self” hypothesis [23]. In homeostasis, negative signals, received from inhibitory receptors, dominate, suggesting that the lack of NK-cell activation does not result from the lack of inhibition, but requires signaling from activating receptors [16]. Stimulation of activating NK-cell receptors overcomes inhibitory signals from inhibitory ones, leading to NK-cell activation and antiviral response (Fig. 1A). Interestingly, apart from CD16 receptor, which mediate ADCC, the cytotoxicity of resting NK cells and their cytokine production might be induced by other activating receptors only in a synergistic way [24]. What is more, NK-cell polarization and degranulation are controlled also by the specific combination of the presence of adhesion molecules, such as ICAM-1 or leukocyte functional antigen-1 (LFA-1) [25].

NK-cell receptors are also involved in recalling their response upon antigen or cytokine re-exposure, which phenomenon established a concept of NK cell “memory” [10]. NK-cell memory-like state is defined by an activation, followed by a return to cell activity baseline, and a highly elevated response following restimulation with the original stimulus. Thus, the types of NK-cell “memory” highly depend on the stimulus, and exhibit different functional characteristics [26]. Nowadays, three different mechanisms of memory-like (or adaptive-like) NK-cells differentiation were established: (i) cytomegalovirus (CMV)-induced, (ii) cytokine-induced, and (iii) liver-restricted memory [26]. In CMV-induced memory, e.g. memory-like NK cells consist of a subset of CD56^dim^CD16^bright^ NK cells with upregulated expression of CD94/NKG2C activating receptor and the maturity marker CD57, accompanied by downregulation of inhibitory receptor CD94/NKG2A [27–30] (Fig. 1B). These cells are present in blood of approx. 30–40% of CMV-seropositive individuals [31]. Murine CMV-induced memory-like NK cells’ expansion was widely studied in the C57BL/6 model and depended on Ly49H receptor which recognizes viral protein m157 expressed on the surface of infected cells [32]. BALB/c and 129/J mice do not express Ly49H receptor and thus exhibit higher susceptibility to murine CMV (MCMV) infection [33].

Finally, NK cells can turn into a state that resembles exhausted T cells, characterized by downregulation of cytotoxicity, cytotoxic proteins production, and/or inhibition of pro-inflammatory cytokine secretion [3, 4, 34–37] (Fig. 1C). This phenomenon comprises a common viral immune evasion mechanism. Many viruses modulate the expression of NK-cell receptors’ ligands on infected cells, which results in either the downregulation of activating receptors or stimulation of inhibitory receptor expression.

## NK-cell receptors in viral infection

**Figure 2.**
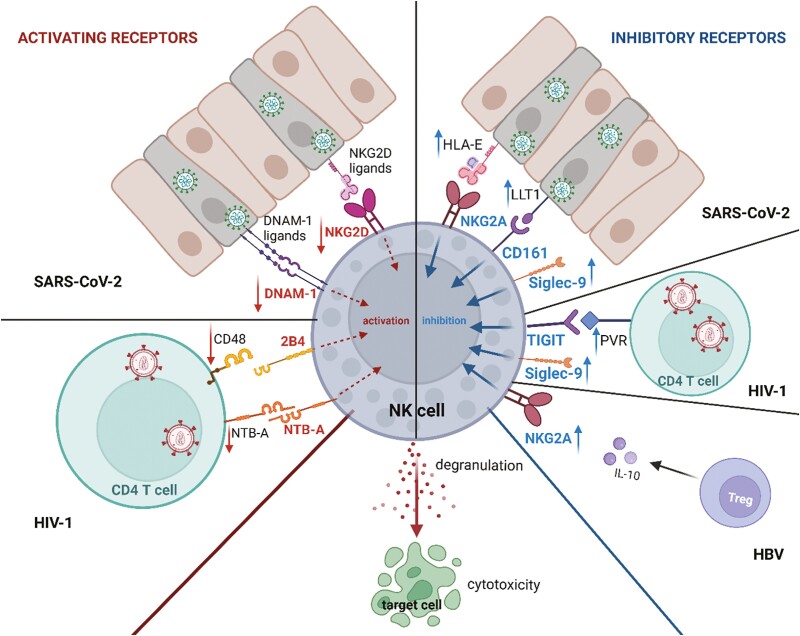
NK-cell receptor-associated inhibition of NK-cell function mediated by aberrant expression of the receptors or ligands. Created with BioRender.com

NK-cell receptor-associated impairment of NK-cell functions was shown in a number of human viral infections, both acute and chronic ones. Viral infection modulates both activating and inhibitory NK-cell receptor functions, limiting NK-cell cytotoxic potential and inducing NK-cell exhaustion state. The alterations of NK- receptor expression along with known mechanisms of their modulation are summarized in Table 1 and presented in Fig. 2.

**Table 1.** Virus-mediated modulation of NK cell receptor expression.

	Virus	Receptor	Receptor-associated modulation in viral infection	References
**Activating receptors**	SARS-CoV-2	NKG2D	Downregulated NKG2D expression resulting from inhibition of NKG2D-L expression by SARS-CoV-2 Nsp-1 protein	Lee [1]
			Upregulated NKG2D ligands, ULBP, expression resulting in downregulated NKG2D expression due to receptor-ligand complex internalization	Wilk [41]
		DNAM-1	Downregulated DNAM-1 expression along with its ligands: PVR and Nectin-4	Hsieh [2]
			Upregulated DNAM-1 ligand Nectin-2 expression resulting in downregulated DNAM-1 expression due to receptor-ligand complex internalization	Wilk [41]
	HBV	NKG2D	Downregulated expression of receptor mediated by upregulated levels of TGF-β1	Sun [42]
		2B4		
	HIV	CD48	Downregulation of CD48 and NTB-A expression mediated by HIV protein viral protein U (Vpu)	Bolduan [47]; Marchitto [48]
		NTB-A		
		CRACC	Upregulated CRACC expression linked with elevated IFN-⍺ levels	O’Connell [49]
	CMV	DNAM-1	DNAM-1 expression corresponds with the stage of CMV infection—stimulation of DNAM-1 ligand’s expression by CMV IE proteins	Pignoloni [50]
**Inhibitory receptors**	SARS-CoV-2	NKG2A	Upregulation of HLA-E expression by Spike protein 1	Bortolotti [3]
		CD161	Downregulated CD161 expression caused by upregulation of its ligand, LLT1 binding and receptor-ligand complex internalization	Lenart [4]
		Siglec-9	Upregulated Siglec-9 –positive CD16^+^CD57^+^NKG2C^+^NKG2A^low^ NK cells in patients; Siglec-9 expression restrain NK cell antiviral response	Saini [5]
	HIV			Adeniji [58]
		TIGIT	Upregulated TIGIT expression on NK cells associated with increased PVR expression on CD4 T cells	Holder [62]
	HBV	NKG2A	Upregulated NKG2A expression mediated by increased IL-10 by Tregs, stimulated by HBeAg	Ma Q [53]

Recent SARS-CoV-2 pandemic provided new insight into NK-cell evasion mechanisms. Clinical studies showed that COVID-19 patients present abnormalities in NK-cell numbers and function. Significantly impaired NK-cell counts and cytolytic activity were observed in COVID-19 patients when compared with healthy controls [38], while patients with normal NK-cell counts were reported to exhibit a faster decline in viral load than patients with low NK numbers [39]. High IL-6 plasma levels, characteristic of severe COVID-19 patients, were associated with the suppression of IFN-γ production in NK cells and inhibition of NK cell normal activity [39]. Increasing evidence suggested that alterations of NK-cell receptors, both activating and inhibitory, might contribute to the dysfunctional status of COVID-19-associated NK cells. Simultaneous blockade of all three natural cytotoxicity receptors (NKp30, NKp44, and NKp46) or 2B4, NKG2D, and DNAM-1 led to a significant increase in virus replication, suggesting that NK-cell-mediated control of SARS-CoV-2 replication in infected target cells requires redundant recognition by activating NK-cell receptors [40] and support an idea of synergistic way of activating NK-receptor functions [24]. Patients who rapidly recovered from SARS-CoV-2 infection presented upregulated cytotoxic activity and had an increased proportion of NK cells expressing DNAM-1 and its paired inhibitory receptor TIGIT. This observation was linked with upregulated expression of ligands of these receptors, poliovirus receptor (PVR) and Nectin-4, that preferentially bound to DNAM-1, stimulating antiviral response [2]. Other studies showed that NK cells isolated form severely ill COVID-19 patients had downregulated surface expression of DNAM-1 and NKG2D, when compared to healthy controls, despite no changes in their gene expression. In parallel, the receptors ligands, Nectin-2 (DNAM-1 ligand), and ULBP proteins, recognized by NKG2D, showed upregulated expression on the peripheral monocytes of COVID-19 patients [41]. The authors suggested that DNAM-1 and NKG2D expression on NK cells is decreased by SARS-CoV-2 due to the ligand overexpression that results in upregulated receptor-ligand complex formation and its internalization. In this study, no alterations of TIGIT receptor, binding Nectin-2, were observed [41]. NKG2D-associate immune evasion mechanism was later revealed in studies of Lee M *et al*. [1], who showed that downregulation of NKG2D ligand is mediated by SARS-CoV-2 non-structural protein 1 (Nsp-1). In agreement with these observations, in our study [4], we observed a significant downregulation of DNAM-1^+^ NK cell proportion after their co-culture with SARS-CoV-2 infected human airway epithelium (HAE), in parallel with an increase of NKG2D^+^ NK cell proportion after co-culture of NK cells with SARS-CoV-2 infected A549^ACE2/TMPRSS2^ cells.

Downregulation of activating NK-cell receptor expression is a common observation in a number of viral infections. Recently, our group showed that children with severe and/or recurrent infections with Herpes simplex virus (HSV) had significantly downregulated expression of activating NK-cell receptors. The expression of CD16 receptor was decreased on CD56^dim^CD16^bright^ NK cells, while both NK-cell subsets exhibited downregulated expression of NKp46, NKp80, NKG2D, and 2B4, evaluated as both the percentage of positive NK cells and the expression level [34]. Decreased NKG2D and 2B4 expression was also associated with HBV infection, along with their intracellular adaptors, DAP10 and SAP, respectively [42]. This observation was linked with upregulated levels of transforming growth factor-beta 1 (TGF-β1) detected in HBV-infected patients and the *in vitro* observation that anti-TGF-β1 antibodies restore NKG2D and 2B4 expression in NK cells [42]. In chronic HCV infection, NK-cells expressing high levels of NKp46 and NKG2D are inversely correlated with liver fibrosis [43]. The expression of NKp46, NKp80, and NKG2D was also shown to be suppressed on NK cells isolated from people with chronic HIV-1 infection, yet was unchanged in HIV controllers. HIV controllers had also a higher proportion of CD56^bright^CD16^dim^ NK cells and increased expression of plasma cytokines including IFN-γ, TNF-α, and IL-12 [35]. Other studies showed that HIV patients present decreased NK-cell expression of NKp30 and NKp46, yet elevated NKp44, NKG2D, and NKp80, accompanied by increased expression of inhibitory NKG2A receptor [36]. Ostrowski *et al*. showed that initially decreased 2B4 expression on NK cells of HIV-infected patients, normalized during highly active antiretroviral therapy [44]. Recent data indicated that the downregulation of NK-cell activating receptors’ expression is mediated by HIV viral protein U (Vpu). Vpu induces CD4 degradation, viral particle maturation, and their release from infected cells [45, 46]. Moreover, Vpu downmodulates CD48 and NTB-A, which act as ligands for 2B4 and NTB-A receptors on NK cells, respectively, contributing to decreased NK-cell degranulation and ADCC [47, 48]. HIV infection was also associated with an increase in NK-cell activating receptor expression, that is CRACC, which was upregulated in CD56^bright^CD16^dim^ NK-cell subset. CRACC upregulation was linked with elevated IFN-⍺ level, suggesting that this receptor plays an important role in the regulation of IFN-⍺–mediated innate immune response during HIV infection [49]. DNAM-1 expression seems to corresponds with the stage of CMV infection, as it switches from upregulation to downregulation upon the progression of the infection from early to late and latent phase. This observation was associated with the stimulation of DNAM-1 ligand’s expression by CMV immediate early (IE) proteins [50].

Viral infections, including SARS-CoV-2, were also associated with modulation of inhibitory receptor expression on NK cells. First reports showed that NK cells isolated from blood and bronchoalveolar lavage fluid of acute respiratory distress syndrome (ARDS) COVID-19 patients presented high levels of PD-1 and NKG2A [51]. NK-cell contact with SARS-CoV-2 Spike protein 1-expressing lung epithelial cells resulted in reduced NK-cell degranulation and was associated with upregulation of NKG2A ligand, HLA-E [3]. Recently, it was shown that NKG2A and KIR2DL1 blockade significantly upregulates the ability of NK cells isolated from COVID-19 patients to lyse SARS-Cov-2 infected cells [52]. NKG2A upregulation was also shown in other viral infections. Patients with active chronic hepatitis B (CHB) had higher percentages of NKG2A-positive NK cells than patients with inactive form of this disease or healthy controls. Furthermore, the proportion of NKG2A^+^ NK cells in CHB patients positively correlated with regulatory T cells (Tregs) numbers and their secretion of IL-10 [53]. This observation was associated with Hepatitis B e antigen (HBeAg), which was shown to induce Treg-mediated IL-10 production that resulted in upregulation of NKG2A expression in NK cells [53]. Similarly, higher expression of NKG2A receptor was observed in NK cells from HCV patients, along with increased production of IL-10 and TGFβ by these cells [54]. However, our *in vitro* studies on NK cells co-cultured with SARS-CoV-2 infected A549^ACE2/TMPRSS2^ cells and HAE cultures did not show any alteration of NKG2A expression on NK cells [4]. We did, though, detect that NK-cell interaction with SARS-CoV-2 infected epithelium mediates the downregulation of expression of other inhibitory receptors, CD161 on NK cells. It was associated with a significant impairment of NK-cell cytotoxicity, a decrease in granzyme B production, and their capacity to control SARS-CoV-2 infection in epithelial cells *in vitro* [4]. The decrease of NK-cell CD161 expression was previously observed in chronic HCV infection [43, 55]. NK-cell function impairment, accompanying the downregulation of CD161, was associated with upregulated expression of CD161 ligand, lectin-like transcript 1 (LLT1) [4]. NK-cell treatment with soluble LLT1 protein resulted in the inhibition of NK-cell cytotoxicity toward K562 cells [4, 56]. Elevated LLT1 levels were shown in patients with respiratory syncytial virus (RSV) infection and associated with elevated proinflammatory cytokine levels, such as type I IFNs, IL-1β, and TNF-α [57], as well as was observed in COVID-19 patients’ sera [4]. However, the mechanism of modulation of LLT1 expression by SARS-CoV-2 is still unknown. Recently, decreased degranulation against SARS-CoV-2 antigen-expressing cells by NK cells isolated from COVID-19 patients was associated with higher glyco-immune checkpoint Siglec-9 expression on NK cells [5]. Siglec-9^+^ NK cells resembled activated and mature CD16^+^CD57^+^NKG2C^+^NKG2A^low^ phenotype and presented higher ADCC levels than Siglec-9-negative NK cells [5]. Moreover, Siglec-9-positive CD56^dim^CD16^bright^ NK cells negatively correlated with HIV viral load [58]. Both reports showed that Siglec-9 expression on highly cytotoxic NK-cell subset restrains their cytolytic functions, and proved that inhibition of this restrain might be achieved by Siglec-9 neutralizing antibodies [5, 58].

Viral infections were also associated with alterations of other inhibitory NK receptors. Chronic viral-induced hepatitis was associated with upregulated expression of TIGIT and TIM-3. Elevated co-expression of these two receptors was observed on NK cells isolated from hepatitis B virus-related hepatocellular carcinoma patients. TIGIT^+^TIM-3^+^ NK cells presented an exhausted and dysfunctional phenotype, including decreased cytotoxic capacity, cytokine production, and proliferation [59]. Likewise, increased TIM-3 expression on NK cells was noted in chronic HBV patients [60], while TIGIT upregulation was detected in HIV-infected patients [61] . High TIGIT expression was linked to inhibited production of IFN-γ by NK cells, while its inhibition restored IFN-γ production [61]. Upregulated TIGIT expression on NK cells in people living with HIV was correlated with higher expression of TIGIT ligand, PVR, on CD4 T cells, when compared to seronegative controls [62]. Upregulation of TIGIT was also indicated to negatively regulate cervical NK cell-mediated immunity to HPV and contribute to the progression of cervical intraepithelial neoplasia [63].

## Perspective

The alterations of NK-cell receptor expression observed in a number of viral infections give the opportunity to develop novel antiviral therapy approaches. The therapeutic effects of NK-based immunotherapy can be achieved by upregulating activating receptor expression on NK cells, their ligands on target cells, or providing the latter in soluble form. This approach is being currently introduced in cancer studies, involving, i.e. stimulation of NKG2D-mediated response [64]. Yet an even more frequent approach is to inhibit immune checkpoints, including NK-cell inhibitory receptors. As an example, an antibody against NKG2A, monalizumab, restoring the cytotoxic functions of CD8 T and NK cells has been introduced in cancer immunotherapy [65]. The data summarized in this review show that many NK-cell receptors might become a potential therapeutic target in novel antiviral therapies. As an example, the blockage of NKG2A, KIR2DL1, Siglec-9, and CD161 enhanced the ability of NK cells to kill SARS-Cov-2 infected cells [4, 5, 52], while disruption of Siglec-sialoglycan interactions stimulated NK-cell-mediated anti-HIV response [58]. However, it is important to bear in mind that the simultaneous blockade of the same receptor, on NK cells and T lymphocytes, might mediate different responses of these two cell populations. As an example, the blockage of LLT1-CD161 axis might enhance NK cell-mediated cytotoxicity [4], yet inhibit T-cell functions [19]. Thus, although modulation of NK-cell receptor activation gives a promising future perspective as an addition to currently used antiviral therapies, a deeper understanding of NK-cell receptor role in viral infections is still needed.

## Data Availability

The manuscript does not contain any original data, as it is a review.

## Abbreviations:

FasL: Fas ligand
GM-CSF: granulocyte-macrophage colony-stimulating factor
HLA: human leukocyte antigen
LFA-1: leukocyte functional antigen-1
MIP-1: macrophage inflammatory protein
NCR: natural cytotoxicity receptors
NK: natural killer
SLAM: signaling lymphocytic activation molecule
TNF-α: tumor necrosis factor
